# An achromatic neutron lens

**DOI:** 10.1038/s41467-026-74925-w

**Published:** 2026-06-29

**Authors:** Mano raj Dhanalakshmi Veeraraj, Di Qu, Hui-Yuan Chen, Silas Strebel, Peng Qi, Anna Fedrigo, Lukas Helfen, Alessandro Tengattini, Matteo Busi, Hongchang Wang, Piero Tranchida, Anders Kaestner, Christian David, Markus Strobl, Joan Vila-Comamala

**Affiliations:** 1 PSI Center for Photon Science, Paul Scherrer Institut, Villigen PSI, Switzerland; 2 Institut Laue-Langevin (ILL), Grenoble, France; 3 University of Grenoble Alpes, CNRS, Grenoble INP, 3SR, Grenoble, France; 4 Institut Universitaire de France (IUF), Paris, France; 5 PSI Center for Neutron and Muon Sciences, Paul Scherrer Institut, Villigen PSI, Switzerland; 6 Diamond Light Source Ltd, Harwell Science and Innovation Campus, Didcot, United Kingdom; 7 SYNOVA S.A., Duillier, Switzerland; 8 Niels Bohr Institute, University of Copenhagen, København, Denmark; 9 DTU Energy, Technical University of Denmark, Lyngby, Denmark; 10 Present Address: XRnanotech GmbH, Villigen PSI, Switzerland

**Keywords:** Imaging techniques, Imaging techniques, Imaging techniques

## Abstract

Neutrons provide exceptional insight into materials, owing to their sensitivity to light elements, isotopic composition, magnetic moments, and high-penetration. However, neutron sources are polychromatic and of low brightness. Neutron optics provides a route to address these limitations by focusing, and to date, various types of neutron optics have been developed based on reflection, refraction, diffraction, and magnetism. Notably, compound refractive lenses and Fresnel zone plates have been demonstrated for imaging, yet their severe chromatic aberration under polychromatic beams has prevented their widespread use and limits progress towards true high-resolution neutron microscopy. Here, we demonstrate an achromatic neutron lens for full-field neutron microscopy. This development overcomes the intrinsic sample-detector distance constraint in pinhole-based radiography. The lens magnification enables the use of efficient detection systems without loss of spatial resolution and establishes a pathway towards high-resolution neutron microscopy. We anticipate the neutron achromat will advance a broad range of neutron methods.

## Abstract (web summary)

Despite the potential of neutron beams in several contexts, applications are currently limited by issues like polychromaticity and low brightness. Here, the authors demonstrate a neutron achromatic lens by combining diamond compound refractive lenses and Ni Fresnel zone plates with microfabrication advances.

## Introduction

Neutron beams are applied to address challenges across a wide range of scientific disciplines, including condensed matter physics^1,2^, materials science^3–6^, energy research^7,8^, biology^9^, nuclear fuel inspection^10^, palaeontology^11^, and cultural heritage investigations^12^. While often conceptually similar to X-ray techniques, neutron methods rely on fundamentally different interactions with matter. X-rays interact primarily with atomic electrons, making them extremely sensitive to high-Z elements. Conversely, neutrons interact with atomic nuclei, providing isotope sensitivity and strong contrast for light elements such as hydrogen, lithium, and boron, which are nearly invisible for X-rays. Owing to their spin, neutrons carry a magnetic moment that enables direct probing of the magnetic properties of materials^13,14^. The high penetration of neutrons into matter enables in situ and operando imaging inside complex sample environments, such as pressure cells and furnaces^15^. Building on these unique properties, neutron imaging^3,4^ is a powerful, non-destructive method for probing the internal structure and composition of materials. Thermal and cold neutrons —with wavelengths ranging from 1 to 10Å— provide an optimal balance of penetration and contrast for imaging applications.

Neutron experiments are usually conducted at large-scale facilities that deliver beams from either a reactor or a spallation source^4^. Compared to synchrotron facilities delivering extremely bright X-ray beams, both types of neutron sources produce beams that are polychromatic and suffer from relatively low brightness. In neutron imaging, these constraints limit the spatial resolution that can be obtained and lead to long acquisition times, hindering further progress in improving spatial resolution. Common neutron imaging instruments use a pinhole-based radiography geometry to record projections by inserting an upstream aperture to collimate the neutron beam and placing the sample in close proximity to an imaging detector to minimise penumbral blurring^16^. The spatial resolution is ultimately limited by the resolution of the detector, which typically consists of a scintillator screen, an optical lens and a pixelated sensor camera. In such a configuration, high-resolution neutron imaging typically requires the use of very thin scintillators^17–19^, at the cost of decreased light output and increased acquisition times. It also requires minimising the geometric unsharpness by employing smaller upstream apertures, which further decreases the neutron flux and increases the acquisition time. To date, spatial resolutions better than 5 μm in neutron imaging have rarely been reported^17–19^ and are generally constrained by the sample-to-detector distance for practical applications in most neutron instruments.

A promising strategy for achieving high-resolution neutron imaging is to transition from a pinhole-based radiography configuration to neutron microscopy, where a neutron lens serves as an objective to form a magnified image of the sample on the detector. In the latter configuration, the imaging performance is effectively decoupled from the geometric constraints inherent to the pinhole-based radiography configuration. This enables upstream sample positioning at extended sample-to-detector distances and within complex experimental environments such as furnaces, cryostats, magnetic field assemblies, and pressure cells. Furthermore, the magnification introduced by the lens allows the use of thicker, higher-efficiency scintillator systems while preserving spatial resolution. To date, Wolter optics^20,21^, CRLs^22–25^, and FZPs^26–28^ have been used as objective lenses in neutron imaging, but the spatial resolutions achieved remain modest, typically around 100 μm. For Wolter optics, further progress critically depends on reducing the figure errors of the reflecting surfaces, making both their fabrication, alignment and assembly highly demanding^21^. In the case of CRLs and FZPs, strong chromatic aberration constrains their performance under the inherently polychromatic conditions of most neutron beamlines.

**Fig. 1:**
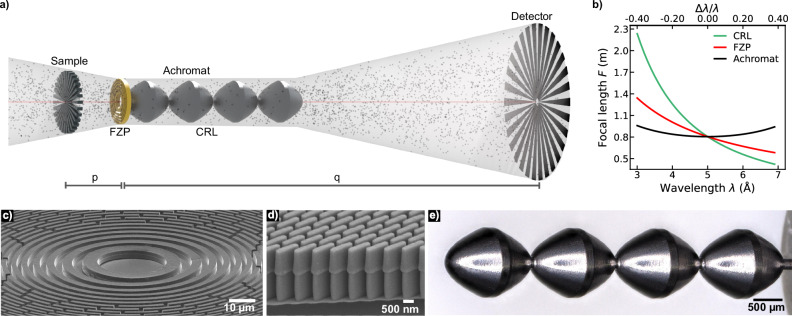
Design and components of the neutron achromat. **a** A neutron achromat employed as the objective lens in a full-field neutron microscope. The achromat is composed of a compound refractive lens (CRL) and a Fresnel zone plate (FZP), and produces a magnified image of the Siemens star on the detector (distance not to scale). **b** Focal length as a function of wavelength for a neutron achromat designed for a central wavelength of 5Å. The curves for an FZP and a CRL with an equivalent focal length are shown for comparison. **c** Scanning electron microscope (SEM) image of a 2-level Ni FZP to be used in a neutron achromat. **d** SEM Cross-section image of a 2-level Ni FZP. **e** Image of the CRL made from a single crystal diamond substrate.

In visible-light optics, a solution to chromatic aberration has existed for over two centuries, consisting of achromatic doublets formed by combining two lenses made from glass materials with different dispersion properties and carefully chosen curvatures. For neutrons, the realisation of achromatic lenses has been proposed several times over the past three decades^22,29,30^ but has not yet been demonstrated experimentally. This optical element, also known as achromat, can be realised by combining a weakly diverging CRL with a strongly converging FZP in close contact, as shown in Fig. 1a. The achromatic behaviour arises from the difference in the dispersion of diffractive and refractive elements: the focal length of a diffractive lens scales inversely with neutron wavelength ${F}_{d} \sim \frac{1}{\lambda }$, whereas it scales inversely with the square of the wavelength ${F}_{r} \sim \frac{1}{{\lambda }^{2}}$ for a refractive lens. By choosing the two elements such that *F*_*r*_ = − 2*F*_*d*_, an achromat with an overall focal length *F*_A_ = 2*F*_*d*_ can be obtained^29,30^. As shown in Fig. 1b, the achromat will have a nearly constant focal length over a wide wavelength range, in contrast to a standalone FZP or CRL. Recently, achromatic and even apochromatic lenses based on similar principles have been demonstrated for X-rays^31,32^.

Here, we present the experimental demonstration of an achromatic neutron lens realised by combining a diamond CRL with a nickel FZP, drawing on advanced micro- and nanofabrication methods originally developed for diffractive X-ray optics. We experimentally verify the achromatic behaviour of the neutron achromat, at neutron wavelengths ranging from 3.5 to 7Å and systematically compare its performance with that of an equivalent FZP with the same focal length. The achromat exhibits significantly reduced chromatic aberration and improved imaging quality across a broad wavelength range. By employing this optical element as an objective lens, we demonstrate a full-field neutron microscope by imaging a resolution target and a representative sample at an extended sample-detector position, establishing a promising route towards realising more efficient high-resolution neutron imaging.

## Results

A neutron achromat was realised and employed as the objective lens to implement a full-field neutron microscope at the NeXT instrument^16^ at the Institut Laue-Langevin, as schematically shown in Fig. 1a. The neutron achromat was formed by combining a converging nickel FZP and a diverging diamond CRL, as shown in Fig. 1c–e. The resulting achromat had an aperture diameter of 1.4 mm and a focal length of *F*_*A*_ = 0.8 m at a neutron wavelength of 5Å. Details about the design, fabrication, experimental setup, data acquisition, and processing are provided in the methods section.

**Fig. 2:**
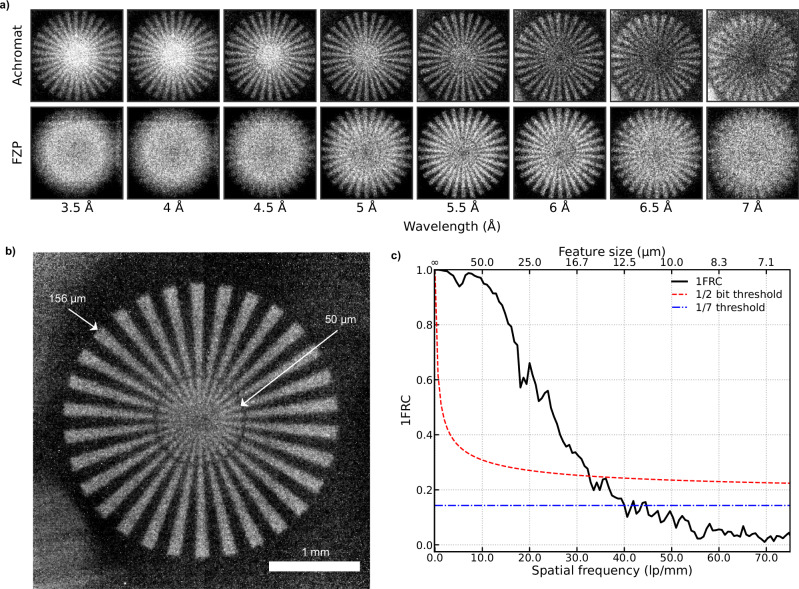
Demonstration of neutron imaging with an achromatic lens. **a** Demonstration of the achromatic behaviour of the neutron achromat and comparison with an equivalent Fresnel zone plate (FZP). A series of neutron images of a 3 mm diameter Siemens star made of Gd was acquired while scanning the neutron wavelength from 3Å to 7Å using a velocity selector with a 15% bandwidth. The neutron achromat and the FZP were used as objective lens positioned at *p* = 880 mm, *q* = 5.9 1 m, in-focus distances corresponding to a neutron wavelength of 5Å and resulting in a magnification of *M* = 6.7 × . The neutron achromat clearly delivers sharper images in a much wider range of wavelengths than the FZP. **b** High resolution magnified image of a 3mm diameter Siemens star acquired by the neutron achromat at 5Å, *p* = 880mm, *q* = 5.91 m and with a longer exposure time of 5400s. **c** The spatial resolution of the neutron image shown in (**b**) was assessed by Fourier ring correlation (FRC), and it is estimated to be below 20 μm.

**Fig. 3:**
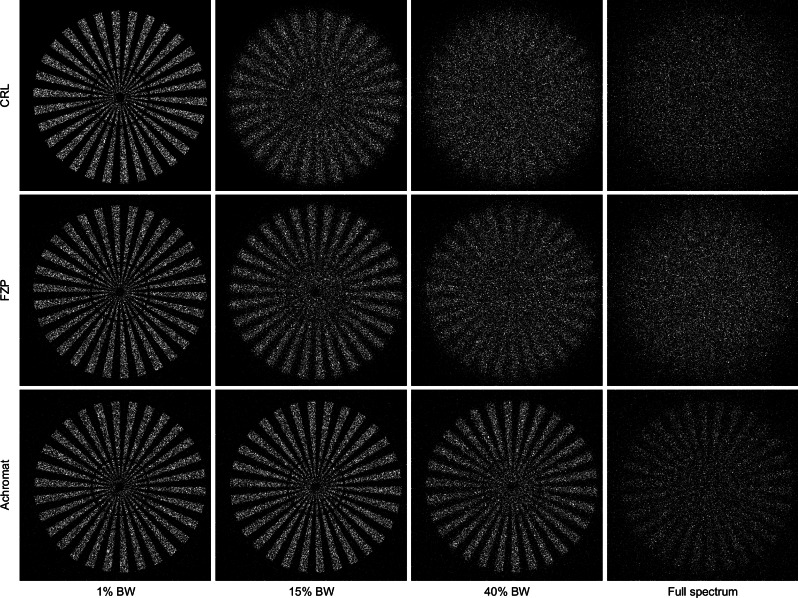
Simulation of bandwidth-dependent neutron imaging with a Fresnel zone plate, a compound refractive lens and an achromat. Simulations of the magnified neutron images of a 3 mm diameter Siemens star object formed by a compound refractive lens, a Fresnel zone plate, and a neutron achromat. All lenses have a diameter of 1.4 mm and a focal length of 0.8 m at a neutron wavelength of 5Å. The bandwidth of the incident neutron beam is varied from 1% FWHM up to the full spectrum of a typical neutron imaging beamline. A total of *n *= 10^8^ neutrons were used for each simulated image. The 15% FWHM bandwidth is a close estimate for the bandwidth of a neutron beam passing through the velocity selector.

The imaging performance of the achromat is compared to that of an equivalent FZP with the same focal length. The wavelength scan shown in Fig. 2a was performed at an object distance *p* = 880mm, by scanning the neutron wavelengths (*λ*) from 3.5Å to 7Å using a velocity selector^33^ with *Δλ*/*λ* ≈ 15% FWHM bandwidth. While the images formed by the FZP are similar to the image formed by the achromat in a narrow range of wavelengths around 5Å, the images formed by the achromat are in focus and have better contrast over a wide range of wavelengths. The observed behaviour closely follows the theoretical curves shown in Fig. 1b. The low signal-to-noise levels of the images at longer wavelengths can be attributed to lower neutron flux at the longer wavelengths, considering that all the images were acquired with identical exposure parameters. To estimate the resolution achievable with the achromat, an image was acquired using the 5Å velocity selector setting, shown in Fig. 2b. Fourier ring correlation (FRC)^34^ is an established method to assess the smallest resolved feature in an image. The smallest feature resolved is estimated by the spatial frequency at which the FRC curve crosses the 1/2-bit threshold and is below 20 μm, as shown in Fig. 2c. As an independent consistency check, a chirped grating was imaged and analysed (shown in Supplementary 1), using vertical averaging of the pixel values to improve the signal-to-noise ratio. In addition, the simulations shown in Fig. 3 were performed to compare the broadband imaging performance of a CRL, an FZP and an achromat. The experimental high-resolution image formed by the achromat shows good agreement with the simulated image using a 15% bandwidth (FWHM) illumination. As a practical demonstration of a real application beyond resolution targets, we imaged a commercially available 10.1mm diameter lithium battery, as shown in Supplementary 2. The battery was positioned at an extended object-detector distance (*q* = 5.91 m), fully compatible with bulky specialised sample environments.

## Discussion

We have experimentally realised an achromatic neutron lens by combining an FZP and a CRL. We demonstrated its achromatic behaviour in a full-field microscope setup, achieving 6.7 × magnification and resolving structures smaller than 20 μm. This work represents an initial proof-of-principle experiment, which can be further scaled up and tailored to match the neutron focusing requirements for various neutron imaging and scattering techniques and adapted for different sample environments and geometries.

The achromat has strong potential to serve as an objective lens that could enable the transition from current pinhole-based radiography geometry to a flexible microscopy geometry with true neutron magnification. The feasibility of the proposed approach is demonstrated by the magnified imaging of a lithium-ion battery at an extended object-detector distance (*q* = 5.91 m), shown in Supplementary 2. Such developments would make it possible to perform high-resolution polarised neutron imaging and in situ/operando study of samples within bulky and complex sample environments without compromising spatial resolution, thereby opening new avenues for dynamic and functional studies of materials under realistic conditions. Drawing from the simulations shown in Fig. 3, compared to an individual FZP or a CRL, the achromat has superior imaging properties and is better suitable to be used for imaging with a velocity selector or generally with less monochromaticity requirement. Notably, the imaging performance of the achromat does not degrade substantially even at 40% bandwidth. The presented achromat can theoretically achieve sub-micron diffraction-limited resolution^35^. The throughput of the achromat can be approximated as the product of the FZP diffraction efficiency and the CRL transmission, yielding a value of  ≈ 32% at 5Å for the lens used in this experiment. The achromat could be further improved by using blazed FZPs to reach higher diffraction efficiency^31,36,37^. In practice, the performance is currently limited by the relative alignment between the FZP and the CRL, neutron shot noise, and the detector system used.

Ultimately, broadband high-resolution neutron microscopy with a lens is limited by two problems: blurring due to chromatic aberration of the lens and blurring due to gravitational dispersion of neutrons of different wavelengths across long flight paths. The achromat has been shown to tackle the first problem. The gravitational blurring can be addressed either by a suitable gravity correction prism^38^ or during the data processing in a time of flight (TOF) based imaging instrument^39^. While no such correction was implemented in this study, no significant degradation of spatial resolution was observed. In future implementations, if required, these adverse effects could be minimised by increasing the magnification through the development of shorter focal length achromatic lenses.

Due to its excellent neutron optical properties, diamond exhibits high refraction and transmission in the cold neutron wavelength range, offering room for further improvements in the CRL design. In this experiment, the theoretical neutron transmission of the divergent CRL at a wavelength of 5Å is approximately 84%. This suggests that the refractive component does not significantly limit the potential for creating an achromat with a shorter focal length. Such a focal length could be achieved either by stacking additional diverging CRLs or by machining paraboloids with a smaller apex radius. However, fabricating a matching FZP to form an achromat would be challenging, as smaller outermost zone widths would be required. Other high-aspect-ratio nanofabrication methods, such as X-ray lithography^40^ (for nickel) or metal-assisted chemical etching^41,42^ (for silicon), have great potential and could be explored to realise the required structures.

Looking forward, a full-field microscope configuration combining Wolter optics as the condenser and an achromat as the objective lens would enhance neutron utilisation efficiency, enabling significantly improved spatial and temporal resolution. However, the beam divergence introduced by such a condenser would not optimally match a single FZP or an achromat, but rather an extended array of FZPs or achromats. Taking advantage of the large divergence and the high flux, parallel multiplexed imaging geometries^25,27,43^ with an array of FZPs or achromats that produce an array of images on the detector will be explored in the future.

In conclusion, the demonstrated achromatic neutron lens represents an important advancement in neutron optical components. By combining precise nano-lithography and micro-machining techniques, this approach enables the fabrication of achromatic neutron lenses that can be customised to meet the specific requirements of various neutron scattering and imaging applications. Moreover, their compact form factor and simple alignment make them particularly attractive, allowing integration into existing neutron instruments and offering a practical and scalable solution for next-generation neutron optics.

## Methods

### Achromat design

A neutron achromat can be formed by combining a diverging CRL of focal length *F*_*CRL*_, where *F*_*CRL*_ ∝ *λ*^−2^, and a converging FZP of focal length *F*_*FZP*_, where the *F*_*FZP*_ ∝ *λ*^−1^, fulfilling the achromat condition *F*_*CRL*_ = − 2*F*_*FZP*_. The resulting achromat will have a focal length *F*_*A*_ = 2*F*_*FZP*_.

The neutron achromat presented was designed to have a focal length *F*_*A*_ = 0.8 m at *λ* = 5Å. The nickel FZP that was a part of the achromat had a diameter *D* = 1.4 mm and outermost zone-width *Δr* = 143 nm with 5.6*μ*m high nickel structures, resulting in a focal length of ${F}_{FZP}=\frac{D\Delta r}{\lambda }=0.4{{{\rm{m}}}}$ with 38% theoretical diffraction efficiency. The diamond CRL that was a part of the achromat had 4 bi-convex paraboloids of diameter *D* = 1.5 mm with an apex radius *R* = 300 μm, resulting in a focal length^44^ ${F}_{CRL}=\frac{R}{2N\delta }=0.8{{{\rm{m}}}}$, where *δ* = 46.6 × 10^−6^ is the decrement in the real-part of the neutron refractive index of diamond, and N is the number of lenses. The theoretical neutron transmission of the diverging CRL at 5Å is  ≈ 84%. The diffraction-limited resolution of the achromat ${\delta }_{r}=\frac{0.610\lambda }{{{{\rm{NA}}}}}=348{{{\rm{nm}}}}$, where ${{{\rm{NA}}}}=\frac{D}{2{F}_{CRL}}$ is the numerical aperture of the achromat.

### Fresnel zone plate fabrication

The FZP in the neutron achromat was a two-level nickel FZP. It was fabricated using a multi-level lithography process^45,46^ following the process flow depicted in Supplementary 3. A 250 μm thick Si substrate was spin-coated with 11% PMMA 950 K in anisole solution at 2050 rpm for 60 seconds and baked at 175 °C for 10 min, resulting in a resist thickness of 2.8 μm. The first-level FZP pattern and the alignment markers were exposed with a 100 keV electron beam using the Raith EBPG 5000 + system. The exposed resist was developed for 22 min in a 7:3 IPA: H_2_0 developer at 5 *°*C. The resist mould was then filled with nickel via pulsed electroplating^47^. The substrate was then spin-coated again with a resist of 2.8 μm thickness and baked for 10 min. The second level of the FZP pattern was then exposed, aligned to the markers, and the process of development and electroplating was repeated. After the second step of lithography, the resist is removed. Bridges were made in the FZP pattern to increase the stability of the resist mould. The resulting FZP had a diameter of 1.4 mm, outermost zone-width *Δr *= 143 nm and nickel structures of 5.6 μm height, resulting in the smallest nano-structures with an aspect ratio of  ≈ 39.

The FZP that was used for comparison was a three-level nickel FZP fabricated on a 2μm resist per level, similar to the above-mentioned process. This FZP had a diameter of 1.2 mm, outermost zone-width *Δr *= 334 nm and nickel structures of 6 μm height, resulting in the smallest nano-structures with an aspect ratio of  ≈ 18.

### Compound refractive lens fabrication and characterisation

The diamond parabolic CRL was fabricated by Synova SA, Duillier, Switzerland, with a Laser MicroJet^®^ turning process. A 2 × 2 × 10 mm^3^ piece of CVD diamond was machined into a 1.5 mm diameter, 8.3 mm long CRL with 4 bi-convex paraboloids with 300*μ*m apex radius as shown in Fig. 1e. The substrate was initially coarsely faceted and progressively turned accurately to match the parabolic profile. At the Beamline B16, Diamond Light Source, Didcot, United Kingdom, X-ray at-wavelength metrology methods^48,49^ were employed to characterise the lens. More details are provided in the Supplementary Table. 1.

### Neutron imaging experimental setup

The neutron imaging experiments were conducted at the NeXT instrument^16^ at the Institut Laue-Langevin, Grenoble, France. The instrument has a 7.5 m long experimental hutch, limiting the maximum distance between the object and the detector. A 35 mm aperture was used in the most upstream position of the beamline. A velocity selector was used to select neutrons of a central wavelength with 15% FWHM bandwidth out of the pink beam from the cold neutron source. A 10 × 10 mm^2^ square slit was used to shape the beam. The full field microscope setup was configured as schematically shown in Fig. 1a. A 3 mm diameter highly absorbing 20 μm thick Gd Siemens star of 30 spokes with features from 156 μm to 8 μm was used as the resolution target for the wavelength scans and the high-resolution image. A 10.1 mm diameter, 5.1 mm tall commercial lithium-ion battery (Varta CoinPower CP1054) with wound electrode design was used as the sample for practical demonstration. The sample was placed  ≈ 300 mm downstream of the beam exit. The lens was positioned at *p* = 880 mm downstream of the sample. The detector was placed at *q* = 5.91 m downstream of the lens in the most downstream position possible within the confines of the beamline, resulting in a magnification of 6.7 × . A high-resolution detector configuration available at the beamline, with a 43.25 × 43.25 mm^2^ field of view and 21.12 μm pixel size, was used with a 20 μm thick Gd_2_O_2_S: Tb/^6^*LiF* scintillator. During the experiment, the detector was binned 2 × 2, hence the effective detector pixel size was 42.24 μm. The virtual pixel size at the object plane, taking into account the neutron magnification, was 6.3 μm.

### Data acquisition and processing

For the wavelength scans shown in Fig. 2a, images were obtained by combining 15 frames with an exposure time of 30s each using the data processing routine detailed below. The high-resolution images shown in Fig. 2b, Supplementary 1 and Supplementary 2 were obtained by combining 180 frames under the same conditions and the same data processing routine.

Multiple raw frames acquired under similar conditions were combined into a single equivalent image using a pixel-wise weighted average combination procedure^50^. The weighting factor for each pixel was determined by the local median absolute deviation (MAD) across multiple frames, which provides a robust estimate of local variability and reduces the influence of outlier values, improving the signal-to-noise ratio while preserving fine structural details.

Following frame combination, flat-field correction was performed to remove the inhomogeneities and artefacts due to the detector response. Then, the dark-current signal recorded by the camera was subtracted to remove baseline offsets. The resulting images were denoised using the BM3D algorithm^51,52^. The filter was applied with the measured standard deviation of the image noise as input, enabling effective denoising while minimising loss of structural information.

In the FRC curves calculated using traditional methods with two independent subsets of data^53^, unconventional artifacts were observed in the high spatial frequency regions. Hence, a single-image FRC^34^ approach, addressed as 1FRC, was used to obtain a more stable and representative resolution estimate.

### Simulations

The imaging simulations shown in Fig. 3 were performed using McStas^54,55^, a Monte Carlo ray-tracing package widely used for modelling and optimising neutron instrumentation. The achromat configuration was implemented by combining the standard *Lens_simple* component with an *FZP_component*. The latter was specifically improved to provide a more accurate treatment of FZP diffraction efficiency. The standalone FZP was modelled using the same *FZP_component,* and the equivalent converging CRL was similarly modelled using the *Lens_simple* with the same diameter of 1.4mm and focal length *F*_*A*_ = *F*_*FZP*_ = *F*_*CRL*_ = 0.8m at the design wavelength 5Å.

Various broadband illumination conditions were generated from the full cold-neutron spectrum by applying a wavelength-dependent triangular transmission function centred at the design wavelength *λ*_0_ = 5Å. For a given fractional base bandwidth *f* = *Δλ*/*λ*_0_, the velocity selector transmission *T*_*f*_(*λ*) was approximated as a symmetric triangle in wavelength space, with its base spanning *λ*_0_ ± *Δλ*/2 and linearly decaying to zero at the edges. In this representation, a base width of *f* corresponds to an effective full width at half maximum (FWHM) bandwidth of *f*/2. By varying *f*, we obtained a series of incident spectra with different effective bandwidths (including a case approximating the 15% FWHM bandwidth of a typical mechanical velocity selector), while the underlying spectral shape of the source was preserved. For each selected bandwidth, the corresponding triangular filter was used to reweight the wavelength distribution in the simulations, as shown in Supplementary Fig. 4.

Including the gravity effects, the broadband imaging performance of the achromat, the FZP and the CRL was simulated in an instrument with *n *= 10^8^ neutrons using the mask component with a binary image of the resolution target positioned such that *p* = 0.92 mm and *q* = 5.87 m at 1%, 15%, 40% (FWHM) bandwidth illumination and with the full cold spectrum. The blurring introduced by neutron events in a thick scintillator is not included in the simulated images.

## Supplementary information

Supplementary information

Transparent Peer Review file

## Data Availability

The raw experimental data were collected during beamtime DIR-369 at the NeXT instrument, Institut Laue-Langevin. The raw data are available from the Institut Laue-Langevin data repository^56^.

## Code availability

The data analysis codes and the simulation codes used during the current study are available from the corresponding author on request.
