# Supplementary material for: An achromatic neutron lens
Source: Nat Commun. 2026 Jun 29;17:8122. doi: 10.1038/s41467-026-74925-w (PMC13458789; doi:10.1038/s41467-026-74925-w)
Supplement: Supplementary file 1 [file 41467_2026_74925_MOESM1_ESM.pdf]

An achromatic neutron lens - Supplementary material

Mano raj Dhanalakshmi Veeraraj[1,*], **Di Qu**[1], **Hui-Yuan Chen**[1], **Silas Strebel**[1], **Peng Qi**[1], **Anna Fedrigo**[2], **Lukas Helfen**[2,3], **Alessandro Tengattini**[2,3,4], **Matteo Busi**[5,6], **Hongchang Wang**[7], **Piero Tranchida**[8], **Anders Kaestner**[5], **Christian David**[1], **Markus Strobl**[5,9,10], **Joan Vila-Comamala**[1]

[1] PSI Center for Photon Science, Paul Scherrer Institut, Villigen PSI, 5232, Switzerland.

[2] Institut Laue-Langevin (ILL), Grenoble, 38042 Cedex 9, France.

[3] University of Grenoble Alpes, CNRS, Grenoble INP, 3SR, Grenoble, 38000, France.

[4] Institut Universitaire de France (IUF), Paris, 75231, France.

[5] PSI Center for Neutron and Muon Sciences, Paul Scherrer Institut, Villigen PSI, 5232, Switzerland.

[6] Present address: XRnanotech GmbH, Villigen PSI, 5232, Switzerland.

[7] Diamond Light Source Ltd, Harwell Science and Innovation Campus, Didcot, OX11 0DE, United Kingdom.

[8] SYNOVA S.A., Duillier, 1266, Switzerland.

[9] Niels Bohr Institute, University of Copenhagen, København, 2100, Denmark.

[10] DTU Energy, Technical University of Denmark, Lyngby, 2800, Denmark.

[*] Correspondence: mano.dhanalakshmi-veeraraj@psi.ch

Supplementary information

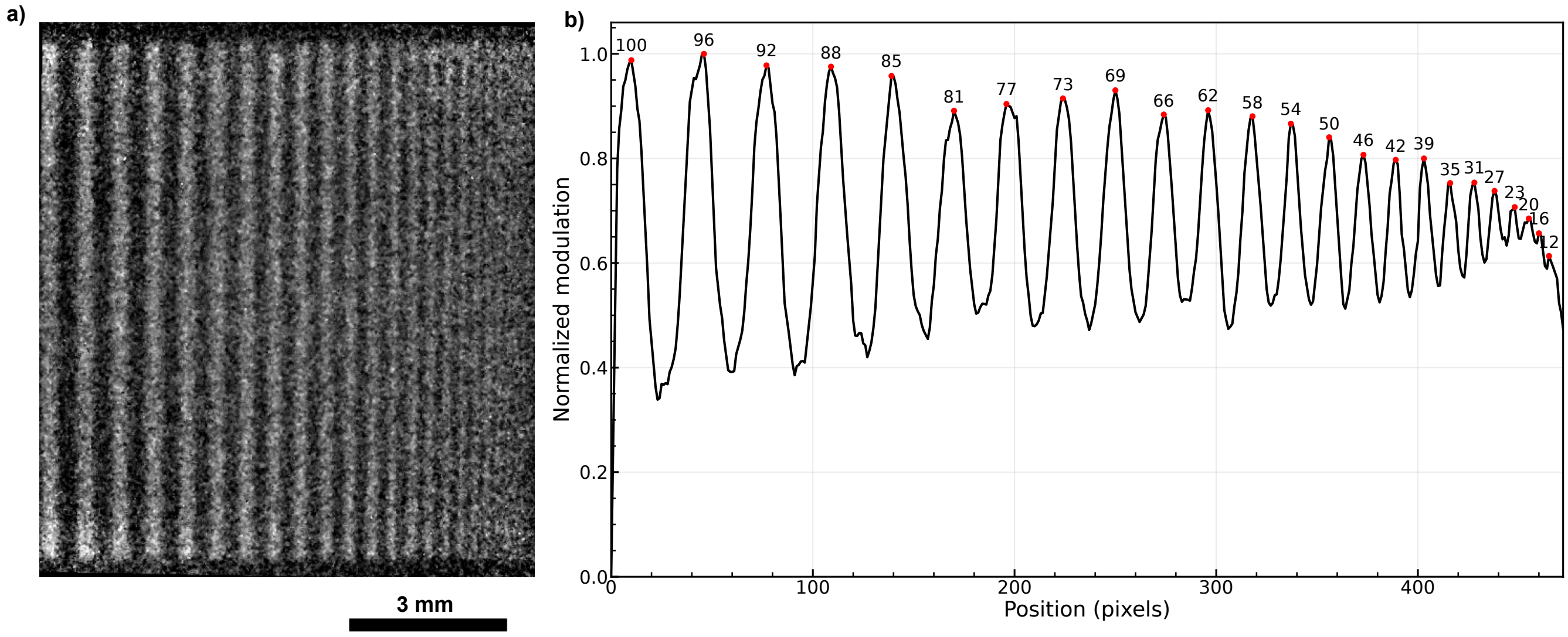

Supplementary Fig.1| High resolution neutron image of a chirped grating. **a**, Magnified neutron image a 3 mm side, 25 line Gd chirped grating with the achromat as the objective lens. The grating has structures from 100 µm to 8 µm line-width. **b**, Vertically pixel-wise averaged line profile of the image presented in (**a**), the line widths corresponding to each modulation are annotated on the top of the peak.

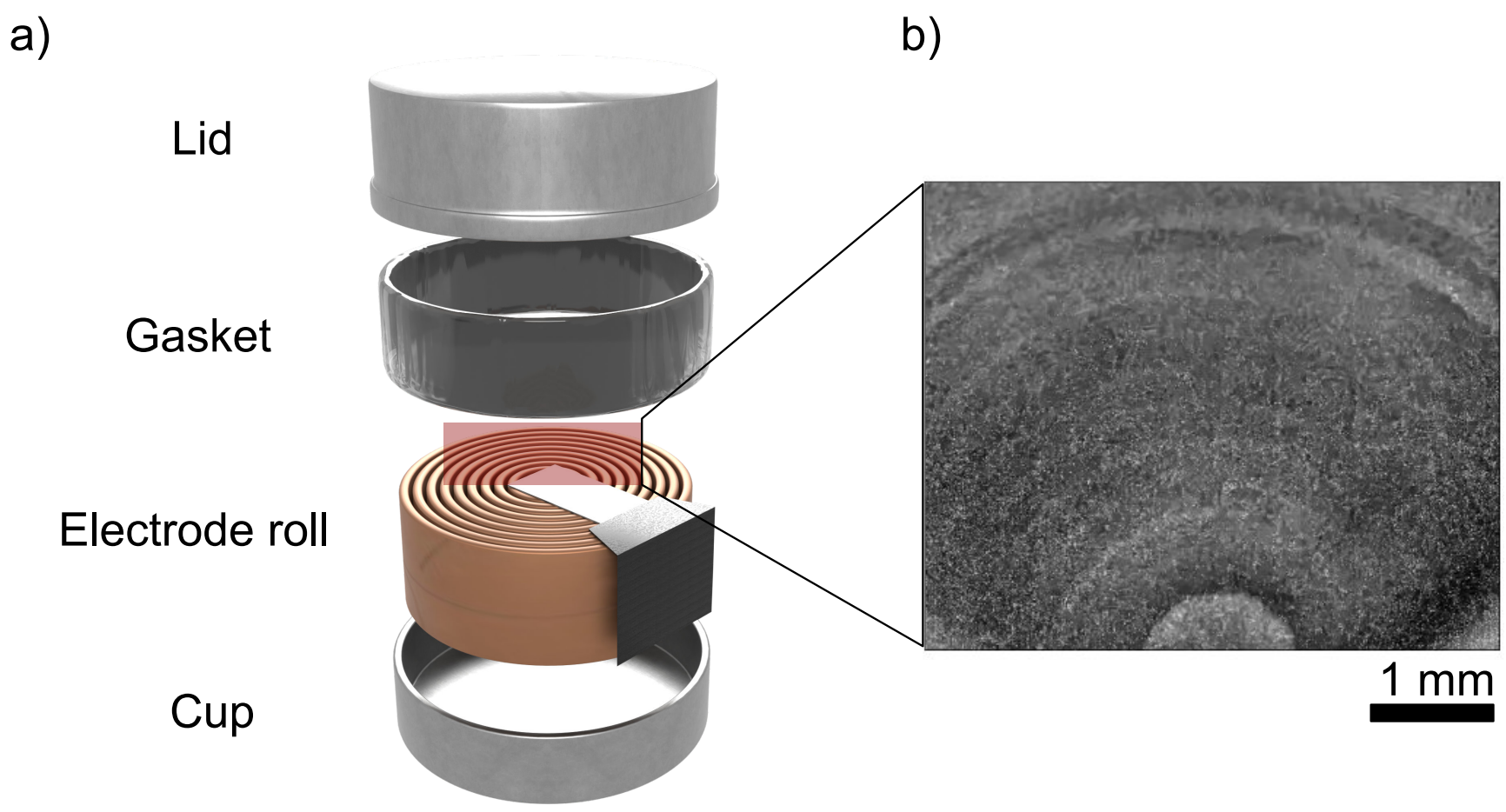

Supplementary Fig.2| Magnified neutron microscope image of a Li battery. **(a)** Schematic of a Varta CoinPower CP1054 Lithium-ion battery, adapted from publicly available manufacturer information. Inside the stainless-steel enclosure, the electrodes, current collectors and the separator are wound together as a roll. The region imaged with neutrons using the neutron achromat objective is marked with the red overlay. **(b)** 6.7× magnified neutron microscope image of a 10.1 mm diameter discharged lithium-ion battery acquired at an extended object–detector distance (q = 5.91 m). Darker contrast corresponds to increased neutron attenuation. Eight concentric windings are resolved within the 4.7 mm field of view, corresponding to an average radial spacing of about 250 µm. The radially symmetric modulation reflects the layered architecture of the wound electrode assembly.

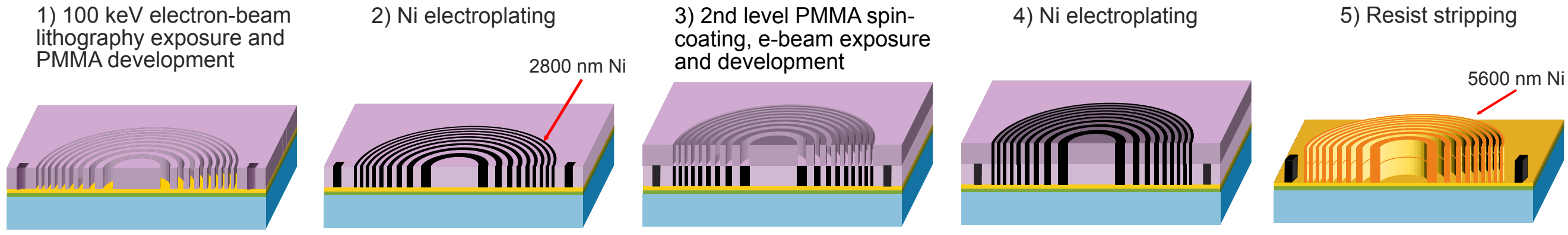

Supplementary Fig.3| Schematic illustration of the two-level FZP fabrication process. A 100 keV electron-beam lithography exposure is performed in 2800 nm-thick PMMA, followed by development to define the first-level zone pattern and the markers. Nickel is electroplated into the developed trenches to form the first 2800 nm-thick Ni level. A second PMMA layer is then spin-coated over, exposed by e-beam lithography aligned to the markers in the first level, and developed. A second Ni electroplating step increases the zone height to a total of $\approx$ 5600 nm. Finally, the remaining resist is removed, yielding the two-level Ni FZP structure.

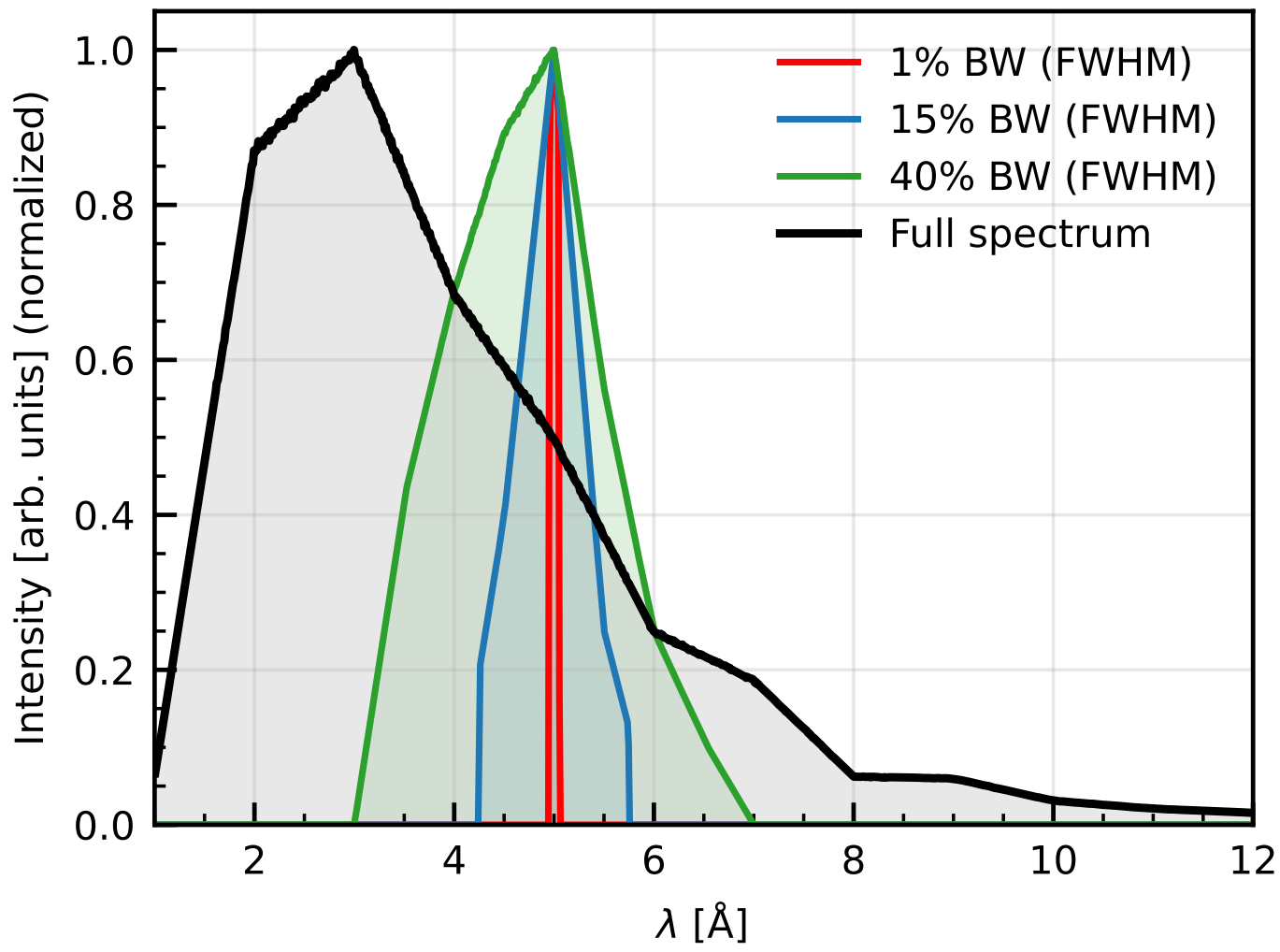

Supplementary Fig.4| Spectra at different bandwidths used as input for the McStas imaging simulations. The *Full spectrum* curve approximates the emission from a cold neutron source typically used in a neutron imaging beamline (NeXT beamline at ILL or ICON beamline at SINQ), while the other curves are obtained by applying a triangular transmission function that mimics a velocity selector centred at a given wavelength, with a specified FWHM bandwidth.

Supplementary Table.1| X-ray characterisation of the diamond CRL. At-wavelength speckle-based metrology was used to quantify the phase-profile slopes of a laser-machined diamond CRL used in the achromat at 15, 20 and 25 keV. The average phase-gradient slope extracted from the speckle displacements was used to determine the experimental X-ray focal length F_{exp}, which is compared with the theoretical X-ray focal length F_{theory} calculated from the corresponding refractive index decrement δ. The percentage error remains below 1% across all energies, indicating only minimal deviations from the ideal parabolic profile.

Energy (keV)	Delta ($\times 10^{-6}$)	Slope of Grad x	Slope of Grad y	Avg slope	F_{exp}	F_{theory}	Error (%)
15	3.24	0.153	0.156	0.1545	11.65	11.57	0.7
20	1.82	0.086	0.088	0.0870	20.69	20.58	0.5
25	1.17	0.055	0.056	0.0555	32.43	32.16	0.8

At-wavelength speckle-based metrology technique[1,2] was used to characterise the phase profile errors of the diamond compound refractive lens. Characterisation was performed at the Beamline B16, Diamond Light Source, Didcot, United Kingdom. In this method, a fine random speckle pattern is placed upstream of the lens, and the resulting speckle images are recorded with and without the lens in the beam. By tracking the small lateral displacements of the speckles introduced by the lens, the slope of the optical phase profile can be determined. In this way, we measured the spatially resolved slope of the phase profile of a laser-machined parabolic diamond CRL. The experiment was performed at 15, 20, and 25 keV, and the focal length of the lenses at 3 different X-ray energies was determined, and the percentage error is calculated from the theoretical focal length at respective energies, shown in Supplementary Table. 1. The very minimal error could be due to a potential deviation from the parabolic phase profile due to fabrication errors.

Supplementary References

[1] Sawhney, K., Wang, H., Sutter, J., Alcock, S. & Berujon, S. At-wavelength Metrology of X-ray Optics at Diamond Light Source. *Synchrotron Radiation News* **26**, 17–22 (2013).

[2] Wang, H., Sawhney, K. & Kashyap, Y. Speckle based X-ray wavefront sensing with nanoradian angular sensitivity. *Optics Express* **23**, 23310–23317 (2015).
