# Supplementary material for: An achromatic neutron lens
Source: Nat Commun. 2026 Jun 29;17:8122. doi: 10.1038/s41467-026-74925-w (PMC13458789; doi:10.1038/s41467-026-74925-w)
Supplement: Supplementary file 2 [file 41467_2026_74925_MOESM2_ESM.pdf]

nature portfolio

Peer Review File

An achromatic neutron lens

Corresponding Author: Mr Mano raj Dhanalakshmi veeraraj

This file contains all reviewer reports in order by version, followed by all author rebuttals in order by version.

Version 0:

Reviewer comments:

Reviewer #1

(Remarks to the Author)
This manuscript reports the experimental realization of an achromatic neutron lens obtained by combining a compound refractive lens with a Fresnel zone plate. The authors demonstrate reduced chromatic defocus under polychromatic illumination and use the device as the objective of a full-field neutron microscope, achieving approximately 20 um spatial resolution and improved image contrast compared to a standalone FZP. Neutron imaging is fundamentally limited by low brightness and large wavelength bandwidth, and chromatic aberration has indeed been one of the main obstacles preventing lens-based neutron microscopy from becoming broadly practical. In this respect, the authors present an impressive achievement which might represents a meaningful technical advance in neutron instrumentation. The manuscript is very well written and technically sound.

However, in its present form the work is best characterized as a proof-of-principle optical component demonstration rather than a demonstrated transition to high-resolution neutron microscopy. The manuscript stops at imaging standard resolution targets, and no application to a real material, device, or in-situ process is presented. As a result, it remains unclear whether the approach presently enables new neutron imaging capabilities in practice, beyond demonstrating the optical component itself. As a consequence, the present results do not yet satisfy the level of generality and demonstrated impact expected for publication in Nature Communications.

Other minor suggestions and comments: 1 The historical overview of neutron lenses is overly long and could be substantially shortened to focus on the specific advance presented here. 2 The claim of “overcoming the flux–resolution trade-off” is not sufficiently supported. The experiment employs a relatively narrow velocity-selector bandwidth and a small effective aperture, both of which reduce flux, and a quantitative comparison with conventional pinhole imaging at comparable acquisition conditions is needed. I think that the demonstration of a substantial application would help solidify this claim.

Reviewer #2

(Remarks to the Author)
In this manuscript entitled "An achromatic neutron lens", the authors developed an achromatic neutron lens by combining a Fresnel zone plate (FZP) and a compound refractive lens (CRL). The method is similar to that in [39] (Sanli et al., 2023), but the authors adopt diamond, which has a very high scattering length density and a very low neutron absorption cross section, for the CRL. Above all, this is the first demonstration of an "acromat" for wide-band neutrons of 3.5-7 Angstrom in wavelength. This is a significant progress, since most neutron sources provide white beams.

Overall, I found the manuscript to be well written, technically solid, and interesting.
It is worth considering for publication after addressing the following minor concerns, which are easy to resolve.

(1) Citation of the following should be included at L174 or somewhere else.
B. Lengeler et al., "Imaging by parabolic refractive lenses in the hard X-ray range", J. Synchrotron Rad. (1999). 6, 1153-1167.

(2) The citation [21] seems incomplete. It should be written, for example;
Cremer, "Introduction to neutron and X-ray optics", Advances in Imaging and Electron Physics vol.172, pp 335-427 (2012).

(3) The method in the experiment to minimize the "gravity effect" should be mentioned.
Wasn't it necessary because the NA of the achromatic lens is high enough?
Or was the effect corrected during the data processing?

(4) Fig.2c should be better explained;
How should we interpret the graph to conclude that the spatial resolution is less than 20 um?

(5) mistyping?
L172: "5.8 um" --> "5.6 um" ? (I see "5.6 um" in L190 and "5600 nm" in Extended Data Fig.1.)
L215: "21.12 um^2" --> "21.12 um" ?

Version 1:

Reviewer comments:

Reviewer #1

(Remarks to the Author)
The authors have adequetly addressed my comments and concerns. The updated and improved manuscript is reccomended for publication.

Reviewer #2

(Remarks to the Author)
The authors have satisfactorily answered all questions of the reviewer and even added the result of an imaging experiment on a lithium-ion battery.
I do not have any further amendments to suggest.

Open Access This Peer Review File is licensed under a Creative Commons Attribution 4.0 International License, which permits use, sharing, adaptation, distribution and reproduction in any medium or format, as long as you give appropriate credit to the original author(s) and the source, provide a link to the Creative Commons license, and indicate if changes were made.
In cases where reviewers are anonymous, credit should be given to 'Anonymous Referee' and the source.
The images or other third party material in this Peer Review File are included in the article's Creative Commons license, unless indicated otherwise in a credit line to the material. If material is not included in the article's Creative Commons license and your intended use is not permitted by statutory regulation or exceeds the permitted use, you will need to obtain permission directly from the copyright holder.
To view a copy of this license, visit https://creativecommons.org/licenses/by/4.0/

We thank the reviewers for their time spent carefully reviewing our manuscript. We have thoroughly read the reviewers' comments, and we have amended the manuscript accordingly. In what follows we provide a detailed response to each one of the reviewers' comments (in **black**), and our responses (in **blue**) and modifications to the text (in **red**).

REVIEWER COMMENTS

Reviewer #1 (Remarks to the Author):

This manuscript reports the experimental realization of an achromatic neutron lens obtained by combining a compound refractive lens with a Fresnel zone plate. The authors demonstrate reduced chromatic defocus under polychromatic illumination and use the device as the objective of a full-field neutron microscope, achieving approximately 20 um spatial resolution and improved image contrast compared to a standalone FZP. Neutron imaging is fundamentally limited by low brightness and large wavelength bandwidth, and chromatic aberration has indeed been one of the main obstacles preventing lens-based neutron microscopy from becoming broadly practical. In this respect, the authors present an impressive achievement which might represents a meaningful technical advance in neutron instrumentation. The manuscript is very well written and technically sound.

We sincerely thank the reviewer for this very positive and encouraging assessment of our work. We appreciate the recognition of the technical significance of the achromatic neutron lens concept and its potential impact on neutron instrumentation. We are grateful for the careful evaluation of the manuscript and the feedback provided.

However, in its present form the work is best characterized as a proof-of-principle optical component demonstration rather than a demonstrated transition to high-resolution neutron microscopy. The manuscript stops at imaging standard resolution targets, and no application to a real material, device, or in-situ process is presented. As a result, it remains unclear whether the approach presently enables new neutron imaging capabilities in practice, beyond demonstrating the optical component itself. As a consequence, the present results do not yet satisfy the level of generality and demonstrated impact expected for publication in Nature Communications.

We thank the reviewer for this careful assessment and criticism. We agree that the original manuscript was primarily focused on demonstrating the realization and quantitative characterization of the neutron achromat using resolution targets, and there was no image of a relevant sample.

To address this important point, we have now included an experimental demonstration of application by imaging a 10.1 mm diameter lithium-ion battery sample (see Extended Data Fig. 4) with neutrons using the neutron achromat as the objective. This measurement was performed at an extended sample–detector distance of 5.91 m, and it demonstrates the feasibility of full-field neutron microscopy with the achromatic lens.

While the present demonstration is not intended as a comprehensive application investigation, it establishes the operational capability of the approach beyond standard resolution targets and provides an example of its applicability. We have also modified the abstract and introduction to reemphasize the merits of transitioning from pinhole-based radiography to microscopy with a lens.

We believe this addition significantly strengthens the manuscript and clarifies the future practical applications of the neutron microscopy method enabled by the neutron achromat.

Other minor suggestions and comments:

1. The historical overview of neutron lenses is overly long and could be substantially shortened to focus on the specific advance presented here.
   We thank the reviewer for this helpful suggestion. In the revised manuscript, we have shortened this section and removed the detailed historical discussion (former Lines 49–56), focusing instead on the specific advance presented in this work. We believe this improves the clarity and the flow of the manuscript.

2. The claim of "overcoming the flux–resolution trade-off" is not sufficiently supported. The experiment employs a relatively narrow velocity-selector bandwidth and a small effective aperture, both of which reduce flux, and a quantitative comparison with conventional pinhole imaging at comparable acquisition conditions is needed. I think that the demonstration of a substantial application would help solidify this claim.
   We thank the reviewer for this important comment. We agree that the wording in the original manuscript could be interpreted as implying that the flux–resolution trade-off has already been quantitatively overcome under the specific experimental conditions presented here. Our intention was instead to highlight the conceptual advantage of the microscopy configuration relative to conventional pinhole radiography.

   To avoid overstatement, we have revised the text to clarify this point.

   The sentence in the abstract now reads:

“This development overcomes the intrinsic constraint on the sample-detector distance imposed by a pinhole-based radiography geometry. The magnification induced by the achromatic neutron lens, enables the use of more efficient detection systems without loss of spatial resolution and establishes a pathway towards high-resolution neutron microscopy”

The sentence in the introduction now reads:

“In the latter configuration, the imaging performance is effectively decoupled from the geometric constraints inherent to the pinhole-based radiography configuration. This enables upstream sample positioning at extended sample-to-detector distances and within complex experimental environments such as furnaces, cryostats, magnetic field assemblies, and pressure cells. Furthermore, the magnification introduced by the lens allows the use of thicker, higher-efficiency scintillator systems while preserving spatial resolution.”

This revised phrasing emphasizes the geometric and conceptual advantages of the microscopy approach, rather than claiming a fully optimized flux performance under the present experimental conditions. It more accurately reflects the demonstrated results and supports our discussion of future extensions of the neutron microscope configuration, including the implementation of a condenser and an array of objectives[1], for which the present work represents an essential first step.

[1] Poulsen, H. F., Andersen, C., Ravinet, N., Vila-Comamala, J., Veeraraj, M. R. D., Knudsen, E. B., Willendrup, P. K., Massahi, S., Christensen, F. E., Ferreira, D. D. M., Strobl, M., David, C., & Kuhn, L. T. (2026). A Neutron Microscope Using a Nested Wolter-I Condenser and a Bank of Diffractive-Refractive Achromatic Objectives. https://arxiv.org/abs/2601.10829v2

Reviewer #2 (Remarks to the Author):

In this manuscript entitled "An achromatic neutron lens", the authors developed an achromatic neutron lens by combining a Fresnel zone plate (FZP) and a compound refractive lens (CRL). The method is similar to that in [39] (Sanli et al., 2023), but the authors adopt diamond, which has a very high scattering length density and a very low neutron absorption cross section, for the CRL. Above all, this is the first demonstration of an "acromat" for wide-band neutrons of 3.5-7 Angstrom in wavelength. This is a significant progress, since most neutron sources provide white beams.

Overall, I found the manuscript to be well written, technically solid, and interesting.
It is worth considering for publication after addressing the following minor concerns, which are easy to resolve.
We thank the reviewer for the careful evaluation of our manuscript and for recognizing the novelty and technical significance of the demonstrated achromatic neutron lens. We have carefully addressed the concerns raised and provide detailed responses below.

(1) Citation of the following should be included at L174 or somewhere else.
B. Lengeler et al., "Imaging by parabolic refractive lenses in the hard X-ray range", J. Synchrotron Rad. (1999). 6, 1153-1167.
We thank the reviewer for this helpful suggestion. The recommended reference has now been included in the revised manuscript at the appropriate location.

(2) The citation [21] seems incomplete. It should be written, for example;
Cremer, "Introduction to neutron and X-ray optics", Advances in Imaging and Electron Physics vol.172, pp 335-427 (2012).
Thank you very much for pointing this out. We have now completed and corrected Ref. [25] in the revised manuscript to include the full bibliographic information.

(3) The method in the experiment to minimize the "gravity effect" should be mentioned.
Wasn't it necessary because the NA of the achromatic lens is high enough?
Or was the effect corrected during the data processing?
We thank the reviewer for raising this important point. In the present experiment, no active gravity correction (e.g., prism-based compensation or Time of Flight-based post-processing) was implemented. The gravitational effect was not explicitly corrected during data processing.

Under the experimental conditions used here, no significant degradation of spatial resolution attributable to gravitational blurring was observed. For better clarity, we have revised the manuscript text accordingly. The relevant sentence now reads:

"While no such correction was implemented in this study, no significant degradation of spatial resolution was observed. In future implementations, if required, these adverse effects could be minimised by increasing the magnification through the development of shorter focal length achromatic lenses."

We believe this clarification addresses the reviewer's concern.

(4) Fig.2c should be better explained;
How should we interpret the graph to conclude that the spatial resolution is less than 20 um?
We thank the reviewer for pointing out that the explanation of Fig. 2c was not sufficiently clear. We have revised the text to clarify how the spatial resolution is determined from the Fourier ring correlation (FRC) analysis.

The revised sentence now reads:

"Fourier ring correlation (FRC) is an established method to assess the smallest resolved feature in an image. The smallest feature resolved is estimated by the spatial frequency at which the FRC curve crosses the 1/2-bit threshold and is below 20 μm, as shown in Fig. 2c"

(5) mistyping?
L172: "5.8 um" --> "5.6 um" ? (I see "5.6 um" in L190 and "5600 nm" in Extended Data Fig.1.)
L215: "21.12 um^2" --> "21.12 um" ?
We thank the reviewer for carefully identifying these typographical inconsistencies. The values have been corrected in the revised manuscript to ensure consistency throughout.
